# Source of dietary fibre and diverticular disease incidence: a prospective study of UK women

**DOI:** 10.1136/gutjnl-2013-304644

**Published:** 2014-01-02

**Authors:** Francesca L Crowe, Angela Balkwill, Benjamin J Cairns, Paul N Appleby, Jane Green, Gillian K Reeves, Timothy J Key, Valerie Beral

**Affiliations:** Cancer Epidemiology Unit, Nuffield Department of Population Health, University of Oxford, Oxford, UK

**Keywords:** diet, dietary fibre, diverticular disease, prospective

## Abstract

**Background:**

Previous prospective studies have found the incidence of intestinal diverticular disease decreased with increasing intakes of dietary fibre, but associations by the fibre source are less well characterised. We assessed these associations in a large UK prospective study of middle-aged women.

**Methods and findings:**

During 6 (SD 1) years follow-up of 690 075 women without known diverticular disease who had not changed their diet in the last 5 years, 17 325 were admitted to hospital or died with diverticular disease. Dietary fibre intake was assessed using a validated 40-item food questionnaire and remeasured 1 year later in 4265 randomly-selected women. Mean total dietary fibre intake at baseline was 13.8 (SD 5.0) g/day, of which 42% came from cereals, 22% from fruits, 19% from vegetables (not potatoes) and 15% from potatoes. The relative risk (95% CI) for diverticular disease per 5 g/day fibre intake was 0.86 (0.84 to 0.88). There was significant heterogeneity by the four main sources of fibre (p<0.0001), with relative risks, adjusted for each of the other sources of dietary fibre of 0.84 (0.81 to 0.88) per 5 g/day for cereal, 0.81 (0.77 to 0.86) per 5 g/day for fruit, 1.03 (0.93 to 1.14) per 5 g/day for vegetable and 1.04 (1.02 to 1.07) per 1 g/day for potato fibre.

**Conclusions:**

A higher intake of dietary fibre is associated with a reduced risk of diverticular disease. The associations with diverticular disease appear to vary by fibre source, and the reasons for this variation are unclear.

**Significance of this study**

**What is already known on this subject?**

- People with a higher intake of dietary fibre appear to have a lower risk of diverticular disease; one study reported variation in the risk of diverticular disease by the source of dietary fibre.

**What are the new findings?**

- We found a significantly reduced risk of diverticular disease with increasing intake of dietary fibre.
- The association with diverticular disease risk varied by the source of fibre, the reduced risk being strongest for cereal and fruit fibre.

**How might it impact on clinical practice in the foreseeable future?**

- A diet high in fibre reduces the risk of diverticular disease.

## Introduction

Forty years ago, Burkitt and his colleagues suggested that diets high in fibre might protect against diverticular disease.^1^ This hypothesis has been supported by results from two prospective studies^2^ ^3^ and findings from one of these suggested that different sources of dietary fibre might have different effects on disease risk.^2^ ^4^ Results from a cross-sectional study^5^ and case-control studies^6^ ^7^ have been inconsistent, but findings from such studies are difficult to interpret since dietary information was recorded at around the time of disease diagnosis, and patients may have changed their diet because of early symptoms, before the formal diagnosis of diverticular disease*.*^8^ Given that a randomised controlled trial examining the long term effect of dietary fibre intake on the incidence of diverticular disease would take many years and might not be feasible, the most practical way to gain better understanding of the association between intakes of dietary fibre and disease risk is in a large prospective study, taking care to allow for possible confounding and reverse causation.

The objective of this study was to assess the association between prospectively-collected information on dietary fibre intake, overall and by the main sources of dietary fibre, in a large cohort of middle-aged UK women.

## Methods

### Participants

The Million Women Study is a population-based prospective study. Between 1996 and 2001, 1.3 million women aged 50–65 years who were invited to attend the National Health Service (NHS) Breast Screening Programme completed a study recruitment questionnaire, which asked about social, demographic and lifestyle factors. Full details of the study protocol and questionnaires are described on the Million Women Study website (http://www.millionwomenstudy.org).^9^ The study was approved by the Oxford and Anglia Multi-Centre Research Ethics Committee and all women gave written consent to be included in the study.

### Assessment of diet

Approximately 3 years after recruitment into the study, women who were still alive were sent a resurvey questionnaire to update information collected at recruitment as well as to ask new questions including those on diet. Participants were asked to report how many times on an average week they consumed a list of approximately 40 common food and beverage items. The mean daily intake of nutrients was calculated by multiplying the frequency of consumption of each food or beverage item by a standard portion size and the nutrient composition of that particular item. The short and long term repeatability of these diet questions and the relative validity of the dietary intake of selected nutrients in comparison with 7-day diet diaries have been reported elsewhere.^10^ The correlation coefficient was 0.62 (r^2^=0.38) for energy-adjusted Englyst dietary fibre (defined as non-starch polysaccharides) intake from the food questionnaire with that from a 7-day diet diary in a randomly-selected sample of 202 women.^10^ Fibre from cereals was estimated from reported consumption of bread, rice, pasta, breakfast cereals, flour, rolled oats, pastry, crackers and biscuits; fibre from fruit from intakes of fresh, and dried and canned fruit. Fibre from vegetables (other than potato) was estimated from intakes of fresh and cooked non-potato vegetables; fibre from potato was estimated from intakes of potatoes, chips and crisps. Participants were also asked whether they consumed a list of white, red or processed meats at least once a week.

### Diverticular disease ascertainment

Each woman's NHS number—a unique personal identifier of NHS records—and date of birth were used to link women to NHS hospital admission data by the Information Services Division of Scottish Morbidity Records (SMR)^11^ and Hospital Episode Statistics (HES) in England.^12^ The dates of hospital admission or of death and associated diagnoses were coded using the WHO Classification of Diseases and Related Health Problems 10th Revision (ICD-10). The main outcome for these analyses was the first hospital admission or cause of death with a mention of diverticular disease (ICD-10 K57, which includes diverticulitis, diverticulosis and diverticulum of the large or small intestine). We have also linked the cohort to data from the NHS Bowel Screening Programme.^13^

### Statistical analysis

Women-years were calculated from the date when diet was reported up to whichever came first: hospital admission with diverticular disease, death, emigration or the last date when hospital records were complete (31 December 2008 for SMR and 31 March 2008 for HES). Cox regression models with attained age as the underlying time variable were used to estimate relative risks (RRs) and 95% CIs of diverticular disease by fifths of total dietary fibre intake, and by fifths of the main dietary sources of fibre (cereals, fruit, vegetables, potatoes). All analyses were stratified by region of residence (10 geographical UK regions) and adjusted for fifths of socioeconomic group (based on Townsend deprivation score),^14^ body mass index (<25, 25–29.9, ≥30 kg/m^2^), height (<160, 160–164.9, ≥165 cm), alcohol consumption (none, <5, 5–10, ≥11 units per week), smoking (never, past, current <10, current 10–14, current 15–19, current ≥20 cigarettes per day), current use of hormone therapy for menopause (yes, no), fifths of total energy intake (kJ) and consumption of meat (no meat, white meat (poultry) only, red (including processed) meat). Data were missing for less than 2% of each of the adjustment variables, and to ensure that the same women were being compared in all analyses the small number with a missing value for each particular variable were assigned to a separate category for that variable and included in the regression analysis.

It is possible that symptomatic diverticular disease may cause abdominal pain, which can result in some people changing their diet, and may lead to ‘reverse causation bias’ whereby the reported diet is a consequence, rather than a cause, of the disease. To minimise this type of bias we excluded 135 172 women (16% of the 866 523 who reported their diet) from these analyses who reported that they had changed their diet over the last 5 years because of their health. Another 6258 women who had a hospital admission with diverticular disease before reporting their diet were excluded, as were a further 35 018 who had a prior cancer registration (except non-melanoma skin cancer), since these conditions may have affected diet.

Reporting of diet on a single occasion is known to be associated with some error. In a prospective study, random misclassification of intakes at baseline or changes in diet during follow-up can result in ‘regression-dilution bias’ whereby the strength of disease risk associated with dietary intakes at baseline would be underestimated.^15^ To allow for such error, we remeasured dietary fibre intake 21 (SD 14) months after baseline in 5059 randomly-selected women (4265 of these women were included in this analysis).^10^ Women were divided into fifths according to their baseline intake of dietary fibre and the mean dietary fibre intake assigned to each fifth was taken using the remeasured values. We calculated the RR per 5 g/day increase in dietary fibre as a trend across the fifths using the remeasured mean dietary fibre intake in each category of dietary fibre at baseline.^15^

For analyses where more than two categories are compared, group-specific variances of log risk were used to calculate the 95% CIs.^16^ Use of this method allows for any two exposure groups to be compared, even if neither group is the reference. For analyses of disease risk per 5 g/day or 1 g/day increment in dietary fibre intake, conventional 95% CIs are used. Heterogeneity in the associations across sources of fibre and subgroups of women was assessed using a standard χ^2^ test.

Various sensitivity analyses were done. To examine the possibility that there was residual reverse causation, we repeated the analyses excluding the first 3 years of follow-up. Analyses were also repeated limiting analyses to diverticular disease separately for ‘complicated’ (ie, diverticula with abscess, bleeding or perforation, ICD-10 code: K570, K572 or K578) or ‘uncomplicated’ disease (ICD-10 code: K571, K573, K575 or K579). Other sensitivity analyses were done to examine the association between dietary fibre intake and diverticular disease in women who were day cases (ie, without an overnight hospital stay) versus women who had required an overnight stay; in women with a current comorbidity using the Charlson index^17^ versus those women without a comorbidity; and in women with a concurrent diagnosis of constipation (ICD-10 code: K590) while in hospital versus those women without a diagnosis of constipation. Diverticular disease is commonly found at colonoscopy in the general population. To assess the robustness of the associations seen here for dietary fibre intake based on hospital admissions for diverticular disease, we examined the association restricting the analysis to women who had a colonoscopy following a positive faecal occult blood test in the NHS Bowel Cancer Screening Programme.

All statistical analyses were performed using Stata statistical software, V.12 (StataCorp, College Station, Texas, USA). Two-sided p values <0.05 were considered statistically significant.

## Results

After excluding women who had changed their diet or had previous diverticular disease or cancer, 690 075 women were eligible for these analyses. At baseline, their mean age was 60 (SD 5) years, and their mean total intake of dietary fibre was 13.8 (SD 5.0) g/day. Table 1 shows the contribution from the main sources of fibre, overall and by fifths of the total fibre intake. Just over two-fifths of the dietary fibre intake was from cereal, and one fifth each from fruit and vegetables other than potato. Correlation coefficients between the various sources of dietary fibre were +0.24 (cereal vs fruit fibre), +0.16 (cereal vs vegetable fibre), +0.01 (cereal vs potato fibre), +0.34 (fruit vs vegetable fibre), −0.12 (fruit vs potato fibre) and +0.06 (vegetable vs potato fibre) (all p<0.001). The correlations between the baseline and remeasured values (n=4265) of dietary fibre were +0.67 for total, +0.67 for cereal, +0.65 for fruit, +0.53 for vegetable and +0.66 for potato fibre (all p<0.001). In the lowest fifth of total fibre intake, the contributions from the various sources of fibre were, respectively, 37%, 16%, 22%, 23% and 2% for cereal, fruit, vegetables, potatoes and other, and in the highest fifth of total intake, these values were, respectively, 42%, 25%, 19%, 11% and 2%.

**Table 1:** Fibre intake (g/day) in the Million Women Study cohort, by fifths of total fibre intake and by the contribution from the main dietary sources to the total

	Fifths of total dietary fibre intake (g/day)					
	<9.6	9.6–12.2	12.2–14.6	14.6–17.6	≥17.6	All women
Number of women	137 951	137 874	138 049	138 078	138 123	690 075
Mean (SD) intake, g/day	7.5 (1.8)	11.0 (0.7)	13.4 (0.7)	16.0 (0.8)	21.0 (3.5)	13.8 (4.9)
Intake from various sources, g/day						% contribution to total
Cereal	2.7	4.5	5.7	7.1	8.9	42%
Fruit	1.2	2.1	2.8	3.5	5.3	22%
Vegetables (not potatoes)	1.7	2.1	2.5	2.9	4.0	19%
Potatoes	1.7	2.1	2.1	2.2	2.4	15%
Other	0.2	0.2	0.3	0.3	0.4	2%

Table 2 shows the characteristics of the women included in the analysis and details of their follow-up, by fifths of total dietary fibre intake. Compared with women in the lowest fifth of total dietary fibre intake (mean remeasured intake=9.5 g/day), those in the highest fifth of intake (mean remeasured intake=18.8 g/day) were from higher socioeconomic groups, slightly taller and leaner, much less likely to smoke, less likely to consume red or processed meat, and had a higher intake of total energy. There were 48 cases of diverticular disease per 10 000 women-years in the lowest fifth of dietary fibre intake compared with 35 cases per 10 000 women-years in the highest fifth. There were similar differences by fifths of cereal, fruit and vegetable fibre intake (see online supplementary eTables 1–3), but there was less variation in these variables across the fifths of potato fibre intake (see online supplementary eTable 4).

**Table 2:** Characteristics of Million Women Study participants according to fifths of total dietary fibre intake

	Fifths of total dietary fibre intake (g/day)					
Categories of fibre intake at baseline	<9.6	9.6–12.2	12.2–14.6	14.6–17.6	≥17.6	All women
Remeasured dietary fibre intake (g/day), mean (SD)*	9.5 (3.8)	11.9 (3.4)	13.7 (3.3)	15.6 (3.7)	18.8 (4.8)	14.2 (5.0)
Characteristics						
Number of women	137 951	137 874	138 049	138 078	138 123	690 075
Mean (SD) age, years	59.7 (4.9)	59.8 (4.9)	59.9 (4.9)	59.9 (4.9)	60.0 (4.9)	59.8 (4.9)
Mean (SD) alcohol intake, units/week†	4.7 (6.3)	4.8 (6.0)	4.7 (5.7)	4.6 (5.6)	4.4 (5.4)	4.6 (5.8)
Mean (SD) height, cm†	161.3 (6.7)	162.0 (6.6)	162.4 (6.6)	162.7 (6.6)	163.2 (6.6)	162.3 (6.7)
Mean (SD) body mass index, kg/m^2^†	26.3 (4.7)	26.2 (4.5)	26.1 (4.4)	25.9 (4.4)	25.6 (4.4)	26.0 (4.5)
Mean (SD) total energy intake (kJ)	6213 (1887)	7435 (1833)	7951 (1893)	8389 (1931)	9228 (2199)	7844 (2197)
Socioeconomic status, lowest third†	44%	35%	31%	29%	28%	33%
Current smokers†	26%	15%	11%	8%	6%	13%
Current users of hormone therapy for menopause†	29%	28%	28%	28%	27%	28%
Consume red or processed meat	87%	88%	88%	87%	84%	87%
Follow-up						
Women-years of follow-up (thousands)	817.1	830.6	837.8	842.3	847.2	4175
New cases of diverticular disease	3943	3629	3452	3300	3001	17 325

*Based on dietary intake in a random sample of 4265 women reported 21 (SD 14) months after baseline.

†Unknown for some women: 1032 for alcohol intake, 8597 for height, 13 632 for body mass index, 5059 for socioeconomic status, 1617 for smoking, 13 676 for hormone therapy use and 5458 for red meat consumption.

**Figure 1:**
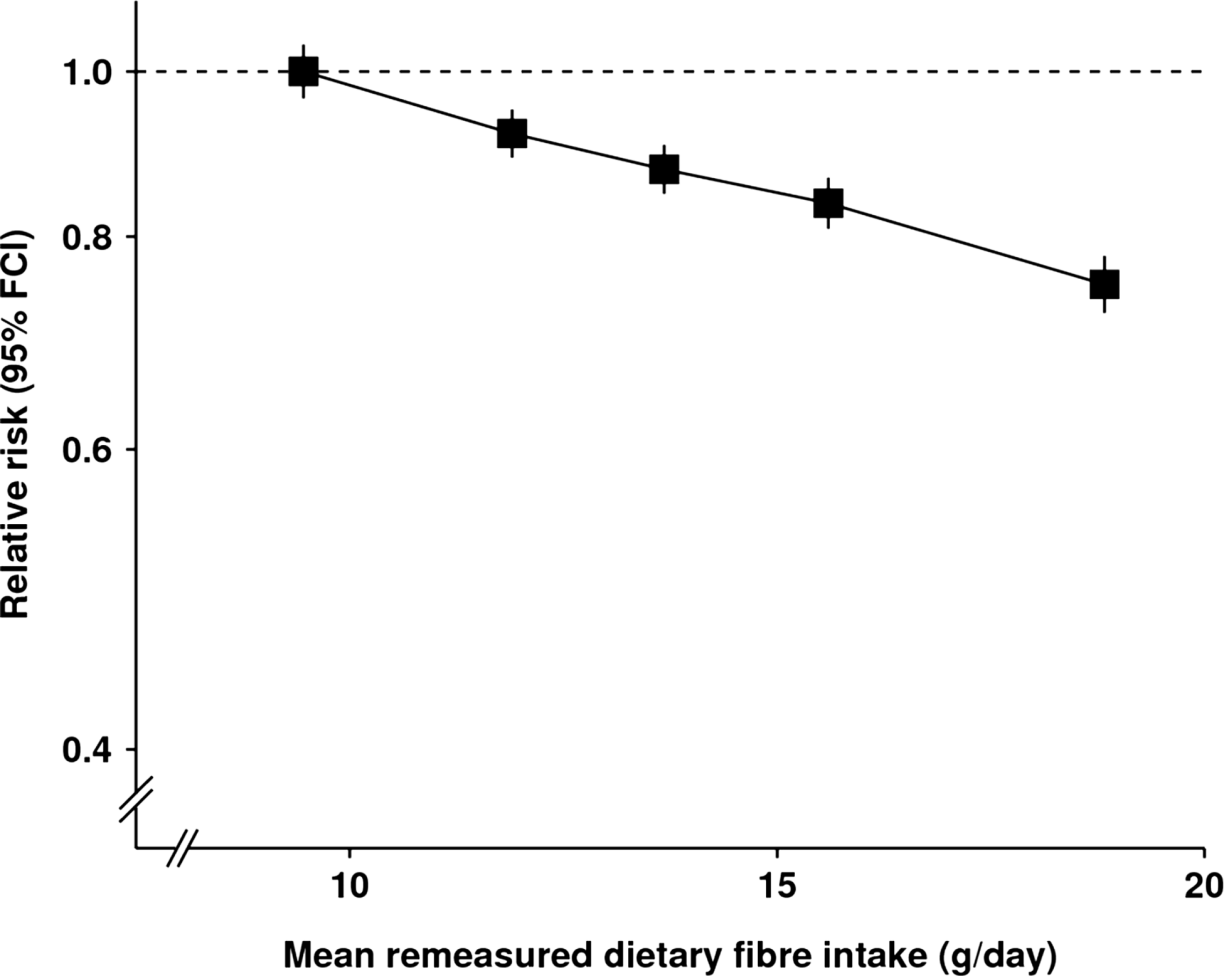
Relative risk of diverticular disease by fifths of dietary fibre intake. Relative risks are stratified by region and adjusted for age, socioeconomic status, smoking, alcohol, body mass index, height, current use of hormone therapy for menopause, total energy intake, and type of meat consumed and are plotted against the mean remeasured dietary fibre intake in each fifth.

After a mean follow-up of 6.0 (SD 2) years, 17 325 study participants were first admitted to hospital or died from diverticular disease (only 21 died from the disease). Table 3 shows the RR of diverticular disease by fifths of dietary fibre intake, overall and by the main sources of fibre intake. There was a strong and highly significant inverse association between total fibre intake and diverticular disease incidence (figure 1). The RR of diverticular disease per 5 g/day intake of total dietary fibre was 0.86 (95% CI 0.84 to 0.88, p<0.0001). The RR for diverticular disease was significantly reduced with increasing intakes of fibre from cereals and fruit, but not for fibre from vegetables or potatoes. The variation between fibre sources was highly significant (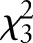
=84.12, p<0.0001; table 4). Mutually adjusting the different sources of dietary fibre for one another attenuated these associations (table 4 and online supplementary table 5).

**Table 3:** Relative risk of hospital admission or death from diverticular disease in the Million Women Study by fifths of dietary fibre intake

	Relative risks and 95% group-specific CIs* by fifths of dietary fibre intake				
	Lowest	2	Middle	4	Highest
Total dietary fibre	1.00 (0.96 to 1.04)	0.92 (0.89 to 0.95)	0.88 (0.85 to 0.91)	0.84 (0.81 to 0.87)	0.75 (0.72 to 0.78)
By source of dietary fibre†					
Cereal	1.00 (0.97 to 1.04)	0.96 (0.93 to 0.99)	0.91 (0.88 to 0.94)	0.86 (0.83 to 0.89)	0.80 (0.77 to 0.83)
Fruit	1.00 (0.97 to 1.03)	0.89 (0.87 to 0.92)	0.88 (0.85 to 0.91)	0.85 (0.82 to 0.88)	0.79 (0.76 to 0.82)
Vegetable (not potato)	1.00 (0.97 to 1.03)	0.94 (0.91 to 0.97)	0.97 (0.93 to 1.00)	0.95 (0.92 to 0.98)	0.94 (0.91 to 0.97)
Potato	1.00 (0.97 to 1.03)	1.02 (0.99 to 1.06)	1.06 (1.03 to 1.10)	1.09 (1.05 to 1.13)	1.14 (1.10 to 1.18)

*Stratified by region and adjusted for age, socioeconomic status, smoking, alcohol, body mass index, height, current use of hormone therapy for menopause, total energy intake and type of meat consumed.

†See online supplementary eTables 1–4 for cut-off values, mean remeasured values and numbers of cases for each category for specific sources of dietary fibre intake.

**Table 4:** Relative risk (RR) of hospital admission or death from diverticular disease in the Million Women Study for total and the various types of fibre, unadjusted and adjusted for intakes of other sources of fibre

	Unadjusted for other sources of fibre		Adjusted for other sources of fibre	
	RR (95% CI)*	p Value	RR (95% CI)	p Value
Total per 5 g/day	0.86 (0.84 to 0.88)	<0.0001		
By source of dietary fibre				
Cereal per 5 g/day	0.81 (0.78 to 0.85)	<0.0001	0.84 (0.81 to 0.88)	<0.0001
Fruit fibre per 5 g/day	0.77 (0.73 to 0.82)	<0.0001	0.81 (0.77 to 0.86)	<0.0001
Vegetable fibre per 5 g/day	0.90 (0.82 to 0.99)	0.035	1.03 (0.93 to 1.14)	0.634
Potato fibre per g/day	1.07 (1.05 to 1.10)	<0.0001	1.04 (1.02 to 1.07)	0.002
Test for heterogeneity by source of fibre	<0.0001		<0.0001	

*Stratified by region and adjusted for age, socioeconomic status, smoking, alcohol, body mass index, height, current use of hormone therapy for menopause, total energy intake and type of meat consumed.

**Figure 2:**
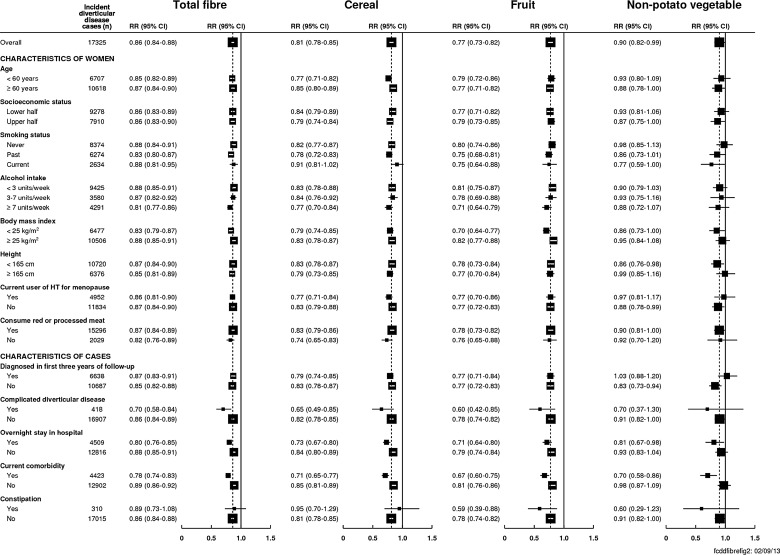
Relative risk (95% CI) of diverticular disease per 5 g/day dietary fibre intake by chosen characteristics. Stratified by region and adjusted for age, socioeconomic status, smoking, alcohol, body mass index, height, current use of hormone therapy for menopause, total energy intake and type of meat consumed (where appropriate). Heterogeneity of trends in relative risk between different subgroups was assessed using a χ² test. Complicated diverticular disease was defined as diverticula with abscess, bleeding or perforation (ICD-10 code: K570, K572 or K578). Current comorbidity was defined using the Charlson index.

The lower risk of diverticular disease for a higher intake of dietary fibre did not vary significantly between subgroups of women defined by their age, socioeconomic status, smoking, alcohol consumption, body mass index, height, use of hormone therapy for menopause or red meat consumption (figure 2). There was no significant association between the consumption of meat and risk of diverticular disease (see online supplementary eTable 6). There was also no significant heterogeneity across the subgroups for intakes of cereal or vegetables (figure 2) or potatoes (see online supplementary eFigure 1); fruit fibre was more strongly associated with the risk of diverticular disease among women with a body mass index less than 25 kg/m^2^ (RR per 5 g/day=0.70, 95% CI 0.64 to 0.77) compared with women with a body mass index of at least 25 kg/m^2^ (RR per 5 g/day=0.82, 95% CI 0.77 to 0.88, p heterogeneity=0.007, figure 2). Results showed similar associations between potato fibre and diverticular disease in women with a low (RR per g/day 1.07, 95% CI 1.04 to 1.10) and a high intake of potato chips (RR=1.06, 0.96 to 1.17, p heterogeneity=0.9).

In a sensitivity analysis that examined the association between dietary fibre and the risk of diverticular disease after excluding the first 3 years of follow-up (10 687 cases included in the analysis), each 5 g/day was associated with an RR for diverticular disease of 0.85 (95% CI 0.82 to 0.88, figure 2). The inverse association with dietary fibre intake was highly significant for both complicated (RR=0.70 95% CI 0.58 to 0.84) and uncomplicated diverticular disease (RR=0.86 95% CI 0.84 to 0.89) with evidence for a stronger association for complicated disease (p heterogeneity=0.021). There was also evidence for an inverse association between dietary fibre and diverticular disease both in women who stayed in hospital overnight and in women who did not (‘day case’ admission), although the association was somewhat stronger in those with overnight stays (p heterogeneity=0.005). A higher intake of dietary fibre was also significantly related to a lower risk of diverticular disease in women with a current comorbidity and without a current comorbidity, with a slightly stronger association among women with a current comorbidity (p heterogeneity <0.001, figure 2). There was no evidence that the inverse association between dietary fibre and diverticular disease differed between women with or without a diagnosis of constipation (p heterogeneity=0.7).

In participants for whom diverticular disease was the first diagnosis or underlying cause of death (*n*=10 673 cases), the risk of diverticular disease for each 5 g/day higher intake was 15% lower (RR=0.85 95% CI 0.82 to 0.88). Since 2006, the NHS Bowel Screening Programme has invited some women in this cohort for faecal occult blood testing. So far, 496 women had diverticular disease diagnosed at colonoscopy and 1039 had a colonoscopy with no abnormality detected following a positive faecal occult blood test. Restricting the analysis to these women yielded an RR of 0.92 (95% CI 0.67 to 1.28) per 5 g/day dietary fibre. Only 39 (8%) of the 496 women had a prior hospital admission for diverticular disease.

## Discussion

In this large cohort of middle-aged women, total intake of dietary fibre was similar to the UK national average for women older than 65 years (13.8 vs 12.5 g/day).^18^ We found a statistically significant reduced risk of diverticular disease with increasing intake of dietary fibre. We also found differences in disease risk by source of the fibre, with significant reductions in risk only with intakes of fruit and cereal fibre. There was little variation in the findings between women from different socioeconomic groups or across other subgroups of women with the exception of fruit fibre by body mass index, although given the large number of tests conducted for this analysis, the role of chance cannot be excluded.

Results from prospective studies consistently show a lower incidence of diverticular disease with a higher intake of total dietary fibre.^2^ ^3^ This contrasts with findings from a recent cross-sectional study^5^ and from some case-control studies,^6^ ^7^ but the results of such retrospective studies are difficult to interpret since patients may have changed their diet because of early symptoms of disease. In all our analyses, women who had changed their diet because of illness in the 5 years before completing the dietary questionnaire were excluded. Furthermore, various sensitivity analyses, including restricting analyses to women diagnosed with diverticular disease more than 3 years after completing the dietary questionnaire, also showed a reduced disease incidence with increasing intake of fibre, making it unlikely that the associations observed here are due to women with early symptoms changing their diet. We corrected for random errors by using remeasured values of fibre intake in our analyses, but we cannot correct for systematic errors in reporting, overall or between different sources of fibre.

Others have suggested that the source or type of dietary fibre may be relevant for diverticular disease.^2^ ^4^ Our results showed significantly reduced risks of diverticular disease for fibre from cereals and fruits but not for fibre from vegetables. The association between diverticular disease and potato fibre has not been reported before, although our finding of increased risks with higher intakes are consistent with reports from the Health Professionals Follow-up Study in the USA that high intake of French fried potatoes was associated with an increased risk of diverticular disease.^2^

The mechanism through which a lower intake of dietary fibre may increase the risk of diverticular disease is hypothesised to be that reduced faecal bulk increases stool transit time;^19–22^ this in turn results in more water being absorbed from the colon and the subsequent formation of harder, smaller stools that may cause straining or constipation. The increased work required to pass these stools may increase the localised intraluminal pressure within segments of the bowel, which is thought to cause herniation (diverticula) of the intestinal wall.^23–25^ There is some evidence from small short-term intervention studies feeding isolated sources of dietary fibre to suggest that stool weight and transit time are affected differentially by food sources such as wheat bran and vegetables.^19–22^ However, it is not clear whether the differences observed in these trials can explain our findings of a stronger association of dietary fibre from cereal and fruit sources. An alternative hypothesis is that dietary fibre alters the bacteria in the gut, which might be an important precursor in developing some of the physiological changes associated with diverticular disease.^26^ ^27^

Data on diverticular disease incidence in the Million Women Study were collected by linkage to hospital records and, if women with lower intakes of dietary fibre were especially likely to develop symptoms such as constipation and be admitted to hospital for their investigation, this could produce a spurious association between dietary fibre intake and diverticular disease. Nevertheless, we found no evidence that the association between dietary fibre and diverticular disease differed between women who received a diagnosis of constipation and those who did not. The study was sufficiently large to examine associations of dietary fibre intake with risk of both uncomplicated (ie, diverticulosis or diverticulitis) and complicated (ie, diverticular disease with abscess, perforation or fistula) disease. Although our results showed a stronger association with complicated than uncomplicated disease, we have no direct data on the validity of the hospital diagnoses, and whether they are sufficiently reliable to distinguish between uncomplicated and complicated disease.^28^ While it is standard practice in the UK to perform imaging (eg, contrast enema) or endoscopy (eg, colonoscopy) to confirm the diagnosis of diverticular disease, we cannot rule out the possibility that a small proportion of women had other disorders, such as IBD.^29^ We also examined the association between dietary fibre and diverticular disease in 496 women with diverticular disease diagnosed at colonoscopy following a positive faecal occult blood test as part of the NHS Bowel Screening Programme (only 8% of these women had a prior hospital admission for diverticular disease). Findings in this small group of women were consistent with the entire cohort but the CIs were wide.

In summary, the evidence from this large prospective study suggests that high intakes of dietary fibre are associated with a reduced risk of diverticular disease, and that this association varies by the source of the fibre.

## Supplementary Material

Web supplement
