# Supplementary material for: Source of dietary fibre and diverticular disease incidence: a prospective study of UK women
Source: Gut. 2014 Jan 2;63(9):1450–6. doi: 10.1136/gutjnl-2013-304644 (PMC4145436; doi:10.1136/gutjnl-2013-304644)
Supplement: Web supplement [file gutjnl-2013-304644-s1.pdf]

eTABLE 1

Characteristics of Million Women Study participants according to fifths of cereal fibre intake

	Fifths of cereal fibre intake (g/day)					
Categories of fibre intake at baseline	< 3.2	3.2-4.7	4.7-6.3	6.3-8.2	≥ 8.2	All women
Remeasured cereal fibre intake (g/day), mean (SD)*	3.3 (2.1)	4.6 (2.2)	5.5 (2.0)	6.8 (2.2)	8.8 (2.8)	5.9 (3.0)
Dietary fibre intake from all other sources (g/day), mean (SD)	6.8 (3.4)	7.7 (3.3)	8.2 (3.3)	8.5 (3.4)	8.9 (3.5)	8.0 (3.5)
Characteristics						
Number of women	137,954	138,379	137,617	138,596	137,529	690,075
Mean (SD) age, years	59.7 (4.8)	59.8 (4.9)	59.8 (4.9)	59.9 (4.9)	60.0 (5.0)	59.8 (4.9)
Mean (SD) alcohol intake, units/week	5.1 (6.6)	4.8 (6.0)	4.6 (5.7)	4.5 (5.4)	4.2 (5.2)	4.6 (5.8)
Mean (SD) height, cm	161.5 (6.7)	162.1 (6.6)	162.4 (6.6)	162.7 (6.6)	163.0 (6.6)	162.3 (6.6)
Mean (SD) body mass index, kg/m^2	26.3 (4.7)	26.2 (4.6)	26.1 (4.5)	25.9 (4.4)	25.6 (4.3)	26.0 (4.5)
Mean (SD) total energy intake, kJ	6,581 (2,042)	7,575 (1,975)	7,963 (2,017)	8,306 (2,039)	8,796 (2,242)	7,844 (2,197)
Socioeconomic status, lowest third	40%	35%	32%	30%	29%	33%
Current smokers	23%	15%	11%	9%	7%	13%
Current use of hormone therapy for menopause	29%	28%	28%	28%	28%	28%
Consume red or processed meat	88%	87%	87%	86%	85%	87%
**Follow-up**						
Women-years of follow-up (thousands)	815.9	827.2	831.3	846.4	854.2	4,175
New cases of diverticular disease	3,783	3,657	3,445	3,278	3,162	17,325

*Based on dietary intake in a random sample of 4265 women reported 21 (SD 14) months after baseline.

eTABLE 2

Characteristics of Million Women Study participants according to fifths of fruit fibre intake

	Fifths of fruit fibre intake (g/day)					
Categories of fibre intake at baseline	< 1.2	1.2-2.0	2.0-3.0	3.0-4.5	≥ 4.5	Overall
Remeasured fruit fibre intake (g/day), mean (SD)*	1.2 (1.0)	2.1 (1.5)	2.9 (1.8)	3.7 (1.8)	5.5 (2.7)	3.1 (2.3)
Dietary fibre intake from all other sources (g/day), mean (SD)	8.9 (3.5)	10.3 (3.4)	11.1 (3.5)	11.6 (3.5)	12.2 (3.9)	10.8 (3.8)
Characteristics						
Number of women	138,529	138,084	137,945	137,679	137,838	690,075
Mean (SD) age, years	59.8 (5.0)	60.0 (5.0)	59.9 (4.9)	59.8 (4.9)	59.7 (4.8)	59.8 (4.9)
Mean (SD) alcohol intake, units/week	4.7 (6.5)	4.5 (5.8)	4.7 (5.6)	4.7 (5.5)	4.5 (5.6)	4.6 (5.8)
Mean (SD) height, cm	161.5 (6.8)	162.0 (6.6)	162.5 (6.6)	162.7 (6.5)	163.0 (6.6)	162.3 (6.6)
Mean (SD) body mass index, kg/m^2	26.2 (4.7)	26.2 (4.5)	26.1 (4.4)	25.9 (4.4)	25.7 (4.4)	26.0 (4.5)
Mean (SD) total energy intake, kJ	7,120 (2,328)	7,733 (2,152)	7,972 (2,104)	8,092 (2,048)	8,306 (2,149)	7,844 (2,197)
Socioeconomic status, lowest third	43%	35%	31%	29%	29%	33%
Current smokers	27%	15%	10%	7%	6%	13%
Current use of hormone therapy for menopause	29%	28%	28%	28%	27%	28%
Consume red or processed meat	89%	89%	88%	86%	81%	87%
**Follow-up**						
Women-years of follow-up (thousands)	828.9	837.9	839.2	837.4	831.7	4,175
New cases of diverticular disease	4,144	3,638	3,437	3,209	2,897	17,325

*Based on dietary intake in a random sample of 4265 women reported 21 (SD 14) months after baseline.

eTABLE 3

Characteristics of Million Women Study participants according to fifths of vegetable (not potato) fibre intake

	Fifths of vegetable fibre intake (g/day)					
Categories of fibre intake at baseline	< 1.5	1.5-2.0	2.0-2.6	2.6-3.5	≥ 3.5	Overall
Remeasured vegetable fibre intake (g/day), mean (SD)*	1.5 (1.0)	2.1 (1.2)	2.5 (1.3)	2.9 (1.3)	4.0 (2.2)	2.5 (1.6)
Dietary fibre intake from all other sources (g/day), mean (SD)	9.1 (4.0)	10.5 (3.8)	11.3 (3.8)	12.0 (3.9)	12.9 (4.4)	11.1 (4.2)
Characteristics						
Number of women	139,731	138,737	136,281	137,395	137,931	690075
Mean (SD) age, years	59.7 (5.0)	59.8 (5.0)	59.9 (4.9)	60.1 (4.9)	59.7 (4.8)	59.8 (4.9)
Mean (SD) alcohol intake, units/week	3.8 (5.4)	4.4 (5.5)	4.7 (5.7)	5.0 (5.9)	5.3 (6.4)	4.6 (5.8)
Mean (SD) height, cm	161.6 (6.7)	162.1 (6.6)	162.3 (6.6)	162.7 (6.6)	162.9 (6.6)	162.3 (6.6)
Mean (SD) body mass index, kg/m^2	26.1 (4.6)	26.0 (4.5)	26.0 (4.4)	26.0 (4.4)	26.0 (4.5)	26.0 (4.5)
Mean (SD) total energy intake, kJ	6,759 (2,118)	7,581 (2,009)	7,983 (2,038)	8,268 (2,066)	8,647 (2,253)	7,844 (2,197)
Socioeconomic status, lowest third	41%	35%	32%	29%	29%	33%
Current smokers	19%	15%	12%	10%	9%	13%
Current use of hormone therapy for menopause	29%	29%	27%	27%	28%	28%
Consume red or processed meat	82%	87%	87%	86%	83%	85%
**Follow-up**						
Women-years of follow-up (thousands)	859.8	849.5	825.7	818.9	821.1	4,175
New cases of diverticular disease	3,864	3,496	3,451	3,314	3,200	17,325

*Based on dietary intake in a random sample of 4265 women reported 21 (SD 14) months after baseline.

eTABLE 4

Characteristics of Million Women Study participants according to fifths of potato fibre intake

	Fifths of potato fibre intake (g/day)					
Categories of fibre intake at baseline	< 1.3	1.3-1.9	1.9-2.4	2.4-2.9	≥ 2.9	Overall
Remeasured potato fibre intake (g/day), mean (SD)*	1.2 (0.9)	1.9 (0.7)	2.3 (0.7)	2.6 (0.7)	3.1 (0.9)	2.3 (1.0)
Mean (SD) fibre intake from all other sources, g/day	11.8 (5.3)	11.8 (4.7)	12.0 (4.6)	11.5 (4.6)	11.3 (4.8)	11.7 (4.8)
Characteristics						
Number of women	175,691	133,651	128,590	118,793	133,350	690,075
Mean (SD) age, years	59.6 (4.9)	59.5 (4.9)	60.0 (4.9)	60.1 (4.9)	60.2 (5.0)	59.8 (4.9)
Mean (SD) alcohol intake, units/week	4.8 (5.9)	4.8 (5.7)	4.8 (5.8)	4.5 (5.7)	4.2 (5.8)	4.6 (5.8)
Mean (SD) height, cm	162.3 (6.7)	162.3 (6.6)	162.5 (6.6)	162.3 (6.6)	162.3 (6.7)	162.3 (6.6)
Mean (SD) body mass index, kg/m^2	25.9 (4.5)	26.1 (4.5)	26.0 (4.4)	26.0 (4.4)	26.2 (4.6)	26.0 (4.5)
Mean (SD) total energy intake, kJ	6,649 (2,000)	7,546 (1,896)	8,014 (1,910)	8,412 (2,004)	9,046 (2,295)	7,844 (2,197)
Socioeconomic status, lowest third	34%	33%	31%	33%	35%	33%
Current smokers	12%	12%	11%	14%	16%	13%
Current use of hormone therapy for menopause	28%	29%	28%	28%	27%	28%
Consume red or processed meat	77%	86%	90%	92%	93%	87%
**Follow-up**						
Women-years of follow-up (thousands)	1,026.0	793.7	776.1	732.6	846.7	4,175
New cases of diverticular disease	3,896	3,088	3,189	3,182	3,970	17,325

*Based on dietary intake in a random sample of 4265 women reported 21 (SD 14) months after baseline.

eTABLE 5

Relative risk of hospital admission or death from diverticular disease in the Million Women Study by fifths of the source of dietary fibre intake further adjusted for all other fibre types.

	Relative risks (RRs) and 95% group-specific CIs (G-S CIs)* by fifths of dietary fibre intake				
	Lowest	2	3	4	Highest
Cereal fibre†	1.00 (0.97-1.04)	0.98 (0.95-1.01)	0.93 (0.90-0.96)	0.89 (0.86-0.92)	0.83 (0.81-0.87)
Fruit fibre	1.00 (0.97-1.04)	0.91 (0.88-0.94)	0.91 (0.88-0.94)	0.88 (0.85-0.91)	0.82 (0.79-0.85)
Vegetable fibre	1.00 (0.97-1.04)	0.96 (0.93-0.99)	1.00 (0.96-1.03)	0.99 (0.96-1.03)	1.00 (0.96-1.04)
Potato fibre	1.00 (0.97-1.04)	1.01 (0.97-1.04)	1.04 (1.00-1.07)	1.04 (1.01-1.08)	1.08 (1.04-1.11)

*Stratified by region and adjusted for age, socioeconomic status, smoking, alcohol, body mass index, height, current use of hormone therapy for menopause, total energy intake, and type of meat consumed.

†See e-tables 1-4 for cut-off values, mean remeasured values and numbers of cases for each category for specific sources of dietary fibre intake

eTABLE 6

Relative risk of hospital admission or death from diverticular disease in the Million Women Study by type of meat.

	Type of meat consumed at least once per week			
	No meat	White meat (poultry) only	Red meat*	*P*
Number of women	21,361	69,474	593,782	
Diverticular disease cases	393	1,510	15,296	
Relative risks (RRs) and 95% group-specific CIs (G-S CIs)†	1.00 (0.91-1.10)	1.07 (1.01-1.12)	1.10 (1.08-1.13)	0.096

*Includes red (beef, lamb, pork, offal) or processed (bacon, ham, sausages, beef burgers/hamburgers) meat.

†Stratified by region and adjusted for age, socioeconomic status, smoking, alcohol, body mass index, height, current hormone therapy for menopause use, total energy intake, and dietary fibre intake.

eFigure 1. Relative risk (95% CI) of diverticular disease per g/day potato fibre intake by chosen characteristics.

	Incident diverticular disease cases (n)	RR (95% CI)
Overall	17325	1.07 (1.05-1.10)
**CHARACTERISTICS OF WOMEN**		
**Age**		
< 60 years	6707	1.07 (1.03-1.11)
≥ 60 years	10618	1.07 (1.04-1.10)
**Socioeconomic status**		
Lower half	9278	1.09 (1.05-1.12)
Upper half	7910	1.06 (1.02-1.10)
**Smoking status**		
Never	8374	1.08 (1.04-1.11)
Past	6274	1.09 (1.05-1.14)
Current	2634	1.02 (0.96-1.08)
**Alcohol intake**		
< 3 units/week	9425	1.08 (1.04-1.12)
3-7 units/week	3580	1.09 (1.03-1.15)
≥ 7 units/week	4291	1.04 (0.99-1.09)
**Body mass index**		
< 25 kg/m^2	6477	1.09 (1.05-1.14)
≥ 25 kg/m^2	10506	1.06 (1.03-1.10)
**Height**		
< 165 cm	10720	1.07 (1.04-1.10)
≥ 165 cm	6376	1.08 (1.04-1.12)
**Current user of HT for menopause**		
Yes	4952	1.10 (1.05-1.15)
No	11834	1.06 (1.03-1.09)
**Consume red or processed meat**		
Yes	15296	1.07 (1.04-1.09)
No	2029	1.13 (1.05-1.21)
**CHARACTERISTICS OF CASES**		
**Diagnosed in first three years of follow-up**		
Yes	6638	1.06 (1.02-1.10)
No	10687	1.08 (1.05-1.12)
**Complicated diverticular disease**		
Yes	418	1.04 (0.89-1.21)
No	16907	1.07 (1.05-1.10)
**Overnight stay in hospital**		
Yes	4509	1.11 (1.05-1.16)
No	12816	1.06 (1.03-1.09)
**Current comorbidity**		
Yes	4423	1.12 (1.07-1.18)
No	12902	1.06 (1.03-1.09)
**Constipation**		
Yes	310	1.03 (0.86-1.23)
No	17015	1.07 (1.05-1.10)

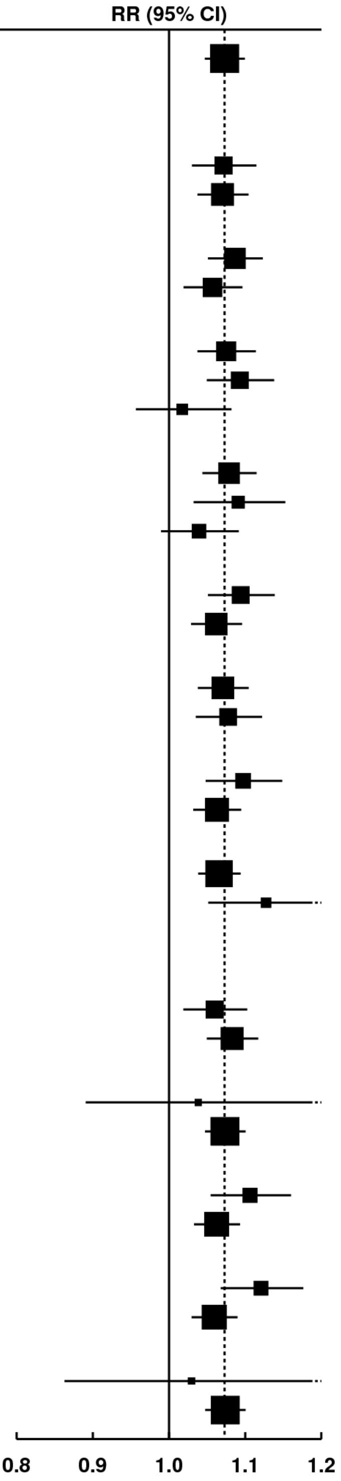

fcddfibreEfig1: 02/09/13
